# Challenges in detecting pre-malignant pancreatic lesions during acute pancreatitis using a serum microRNA assay: a study based on *Kras^G12D^* transgenic mice

**DOI:** 10.18632/oncotarget.8148

**Published:** 2016-03-17

**Authors:** Xiafei Hong, Jie Zhang, Qiao Wu, Wenze Wang, Adam Yongxin Ye, Wei Song, Hongmei Dai, Xianze Wang, Fan Wu, Lei You, Wenming Wu, Yupei Zhao

**Affiliations:** ^1^ Department of General Surgery, Peking Union Medical College Hospital, Chinese Academy of Medical Sciences and Peking Union Medical College, Beijing 100730, China; ^2^ Department of General Surgery, China-Japan Friendship Hospital, Beijing 100029, China; ^3^ Department of Hepatobiliary Surgery, Beijing Chaoyang Hospital, Capital Medical University, Beijing 100020, China; ^4^ Department of Pathology, Peking Union Medical College Hospital, Chinese Academy of Medical Sciences and Peking Union Medical College, Beijing 100730, China; ^5^ Center for Bioinformatics, Peking University, Beijing 100871, China; ^6^ Department of Biochemistry and Molecular Biology, State Key Laboratory of Medical Molecular Biology, Institute of Basic Medical Sciences Chinese Academy of Medical Sciences, Peking Union Medical College, Beijing 100005, China

**Keywords:** microRNA, Kras^G12D^, pancreatitis, PanIN, miR-210

## Abstract

Caerulein-induced acute pancreatitis accelerates the progression of pancreatic intraepithelial neoplasia (PanIN) lesions in a pancreas-specific *Kras^G12D^* mouse model. The purpose of this study was to explore whether serum microRNAs (miRNAs) can serve as sensitive biomarkers to detect occult PanIN in the setting of acute pancreatitis. Serum miRNA profiles were quantified by an array-based method and normalized by both Variance Stabilization Normalization (VSN) and invariant methods. Individual miRNAs were validated by TaqMan real-time PCR with synthetic spike-in *C. elegans* miRNAs as external controls. Serum miRNA profiles distinguished *Kras^G12D^* mice with pancreatitis from wild-type mice without pancreatitis, but failed to differentiate *Kras^G12D^* mice with pancreatitis from wild-type mice with pancreatitis. Most individual miRNAs that increased in *Kras^G12D^* mice with pancreatitis were not significantly different between *Kras^G12D^* mice without pancreatitis and wild-type mice without pancreatitis. Mechanistically, Gene Set Enrichment Analysis (GSEA) of the mRNA array data and immunohistochemical assays showed that caerulein-induced acute pancreatitis involved acinar cell loss and immune cell infiltration, which might contribute to serum miRNA profile changes. This study highlighted the challenges in using sensitive serum miRNA biomarker screening for the early detection of pancreatic malignancies during acute pancreatitis.

## INTRODUCTION

Pancreatic cancer is a leading cause of death in both the United States, with an estimated 53,070 new cases and 41,780 deaths annually [1], and in the People's Republic of China [2]. Despite advances in cancer therapy in recent decades, the five-year survival rate for pancreatic cancer is only 7.2% [3]; however, the five-year survival is higher (27.1%) for patients with localized disease at the time of diagnosis, highlighting the necessity of diagnosing pancreatic cancer early. Pancreatic intraepithelial neoplasia (PanIN) lesions represent a pre-malignant step during pancreatic tumourigenesis [4]. A recent study showed that acute pancreatitis was associated with an increased risk of pancreatic cancer, and the lag period was approximately two years [5]. In addition, patients with a history of pancreatitis are at high risk for subsequent pancreatic cancer [6–8]. Thus, non-invasive screening of patients with acute pancreatitis episodes or a history of pancreatitis is an attractive strategy for detecting PanIN.

Here, we focused on whether non-invasive biomarkers are suitable for detecting PanIN in the setting of acute pancreatitis. Because it is impractical to know *a priori* whether a patient has a PanIN lesion during acute pancreatitis, a pancreas-specific *Kras^G12D^* mouse model was used, wherein PanIN lesions develop during acute pancreatitis induced by intra-abdominal injections of caerulein, a cholecystokinin analogue. For the biomarkers, we focused on microRNAs (miRNAs), which are 22-nucleotide RNA molecules [9] that may serve as candidate diagnostic biomarkers for pancreatic cancer due to their excellent stability in circulating body fluids, including whole blood [10–12], plasma [13], serum, and saliva [14]. We also investigated the pathological process responsible for caerulein-induced acute pancreatitis and its potential influence on circulating miRNAs.

## RESULTS

### Transgenic mouse model recapitulating PanIN lesions during acute pancreatitis

**Figure 1:**
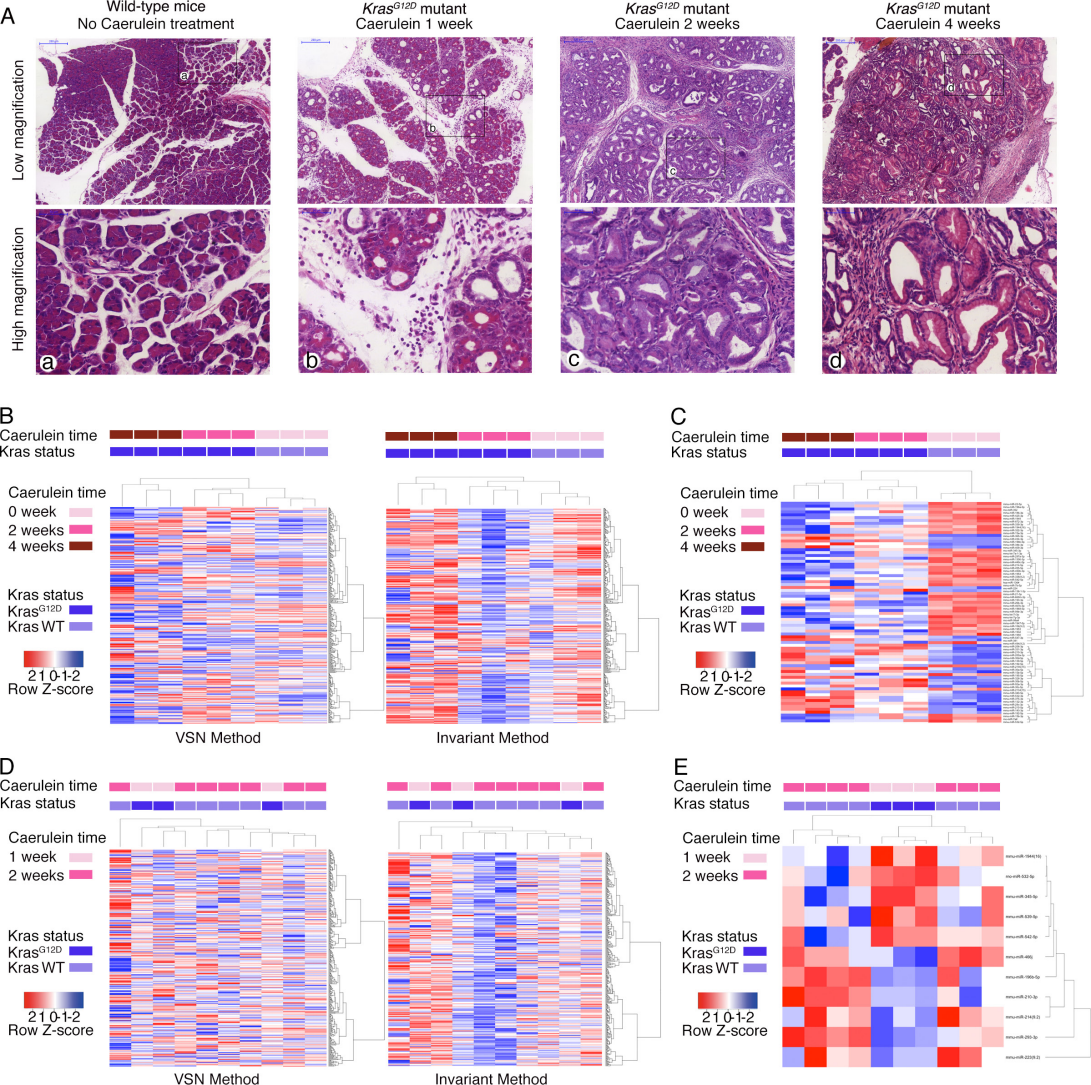
Caerulein-induced acute pancreatitis causes pathological changes and altered serum miRNA profile (**A**) H & E staining of pancreas of wild-type mice without acute pancreatitis, *Kras^G12D^* mice with 1-, 2- and 4-week of caerulein injection (from left to right). Upper panels were in low magnification and lower panels were the corresponding high magnification images. (**B**) Heatmap of serum miRNA changes among wild-type mice without acute pancreatitis, and *Kras^G12D^* mice with 2- and 4-week of caerulein-induced pancreatitis (both VSN and invariant normalization methods). (**C**) Heatmap of significantly altered serum miRNA among wild-type mice without acute pancreatitis, and *Kras^G12D^* mice with 2- and 4-week of caerulein-induced pancreatitis. (**D**) Heatmap of serum miRNA changes between wild-type mice with 2-week of caerulein-induced pancreatitis, and *Kras^G12D^* mice with 1-week of caerulein-induced pancreatitis (both VSN and invariant normalization methods). (**E**) Heatmap of significantly altered serum miRNA between wild-type mice with 2-week of caerulein-induced pancreatitis, and *Kras^G12D^* mice with 1-week of caerulein-induced pancreatitis.

The present study used two mouse models: *Kras^G12D^* mice that harbour a pancreas-specific activating *Kras^G12D^* mutation, and wild-type mice that express wild-type *Kras* in the pancreas. Caerulein was intra-abdominally injected into one-month-old mice for 1, 2, or 4 weeks to induce acute pancreatitis, and the mice were subsequently sacrificed upon completion of caerulein dosing (Figure 1A). As the time of caerulein injections increased, the degree of pancreatitis worsened, and PanIN lesions progressed. Typically, wild-type mice showed normal pancreatic histology, with predominantly normal-looking acinar cells (Figure 1A). *Kras^G12D^* mice with 1-week pancreatitis presented with normal acini and acinar-to-ductal metaplasia. No obvious PanIN lesions were observed at this stage (Figure 1A). In contrast, *Kras^G12D^* mice with 2- or 4-week pancreatitis presented with PanIN (mostly PanIN-1) lesions and interstitial fibrosis (Figure 1A).

### Serum miRNAs can differentiate *Kras^G12D^* mice with pancreatitis from wild-type mice without pancreatitis

Serum samples from six *Kras^G12D^* mice with either 2- or 4-week pancreatitis and three wild-type mice without caerulein treatment were quantified using miRNA arrays. Overall, 366 of 768 miRNAs were detected in all serum samples. The serum miRNA profile could differentiate the genotypes and different durations of caerulein injections into three distinct clusters by both Variance Stabilization Normalization (VSN) and invariant methods (Figure 1B). The VSN and invariant methods detected 85 and 108 miRNAs with significantly different expressions, respectively. Seventy-six significantly changed miRNAs were detected by both methods, with a kappa index of agreement of 0.71. Of these 76 miRNAs, 30 were increased, and 46 were decreased (Supplementary Table 1). These 76 significantly changed miRNAs revealed the capacity for differentiating the various groups (Figure 1C).

### Serum miRNA profiling cannot differentiate *Kras^G12D^* mice with pancreatitis from wild-type mice with pancreatitis

We next asked whether the affected miRNAs could differentiate mice with PanIN lesions during acute pancreatitis. Serum samples from three *Kras^G12D^* mice with 1-week pancreatitis and seven wild-type mice with 2-week pancreatitis were quantified using miRNA arrays. Overall, 362 of 768 miRNAs were detected in all serum samples. However, the serum miRNA profiles could not differentiate the two groups by either normalization method (Figure 1D). The invariant and VSN methods detected 19 and 18 significantly changed miRNAs, respectively. Eleven significantly changed miRNAs were detected by both methods, with a kappa index of agreement of 0.57. Six of these miRNAs were decreased, and five were increased (Supplementary Table 2). These eleven significantly changed miRNAs could not be used to distinguish the two groups (Figure 1E). Interestingly, four of the 11 significantly changed miRNAs (mmu-miR-196b-5p, mmu-miR-210-3p, mmu-miR-1944(16), and mmu-miR-542-5p) were also identified in the previous experiment.

### Increased miRNAs in serum likely originate from the pancreas of *Kras^G12D^* mice with pancreatitis

**Figure 2:**
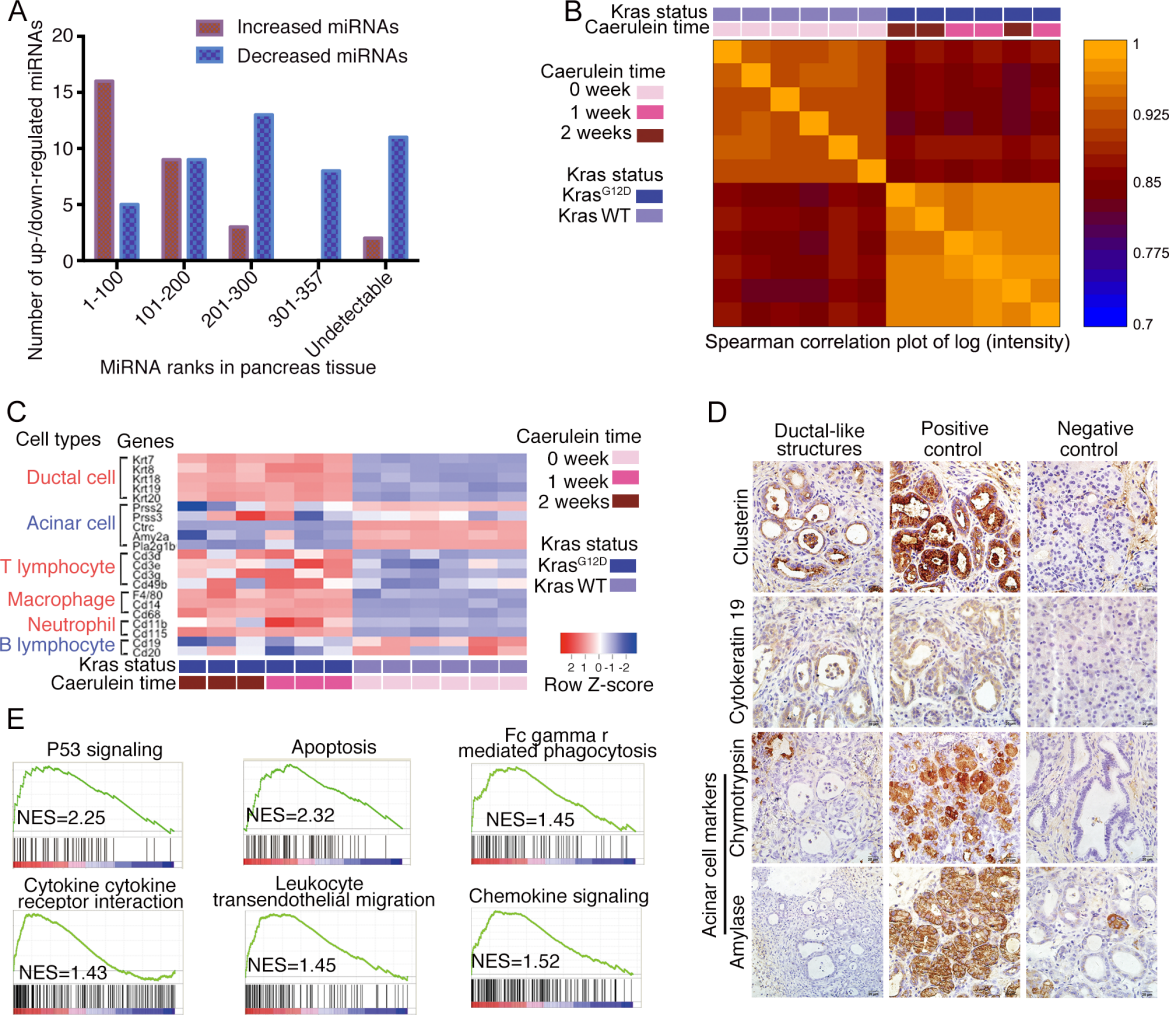
Serum miRNA alterations originate from pancreas (**A**) Significantly increased/decreased serum miRNAs and their ranks in pancreas tissue. (**B**) Spearman correlation plot for mRNA array assay of wild-type mice without acute pancreatitis, *Kras^G12D^* mice with 1- and 2-week of caerulein injection. (**C**) Heatmap of acinar/ductal/immune cell related genes' expressions, data from mRNA array. (**D**) Immunostaining for ductal-like structure. (**E**) Gene Set Enrichment Analysis for enriched pathways in *Kras^G12D^* mice with acute pancreatitis.

We speculated that the increased miRNAs in the serum from the *Kras^G12D^*-pancreatitis group might originate from the pancreas. The miRNA profiles of whole pancreas lysates were quantified in two *Kras^G12D^* mice with 4-week pancreatitis. In total, there were 409 and 394 detectable miRNAs in these two mice; 357 miRNAs were detected in both tissue lysates, and 446 were detected in at least one of the tissue lysates. Over half (16/30) of the increased serum miRNAs in *Kras^G12D^* mice with pancreatitis ranked in the top one hundred miRNAs in the tissue samples. Meanwhile, the decreased miRNAs showed a relatively even distribution in the tissue samples (Figure 2A). These data suggested that the pancreas is the likely source of the increased miRNAs in serum.

### Acinar cell loss and immune cell infiltration occur during pancreatitis in *Kras^G12D^* mice

To ascertain the underlying pathological mechanisms responsible for the circulating miRNA profile changes, the global gene expression profiles in whole pancreas lysates from three 1-week and three 2-week *Kras^G12D^* mice with pancreatitis and six wild-type age-matched mice without pancreatitis were quantified (Data available from GEO Database: Series No. GSE77983). Spearman's correlation analysis demonstrated that the mRNA transcripts could differentiate *Kras^G12D^* mice from wild-type mice but could not differentiate *Kras^G12D^* mice with 1-week or 2-week pancreatitis (Figure 2B), indicating that similar mRNA profile changes occurred in these mice. A heatmap showed that the expression of ductal cell-related genes (*Krt7, Krt8, Krt18, Krt19*, and *Krt20*) was significantly higher and that of acinar cell-related genes (*Prss2, Ctrc, Amy2a*, and *Pla2g1b*) was significantly lower in the *Kras^G12D^* pancreatitis group, reflecting shifting dynamics from the acinar cell compartment to the ductal-like cell compartment (Figure 2C). The ductal-like structures expressed a de-dedifferentiated acinar marker (clusterin) and a ductal marker (cytokeratin 19) but lacked mature acinar markers (amylase and chymotrypsin) by immunostaining (Figure 2D). The expression of genes associated with T lymphocytes and macrophages, but not with B lymphocytes, was higher in the *Kras^G12D^* group, indicating that specific immune cell infiltration occurred during the first two weeks of caerulein-induced pancreatitis (Figure 2C). Acinar cell loss and the active participation of immune cells during pancreatitis were also observed by Gene Set Enrichment Analysis (GSEA). Of the twenty most enriched pathways, two (p53 signalling and apoptosis) are involved in acinar cell loss [15], and four are associated with immune cell infiltration (Figure 2E). Pancreatitis promotes the acinar-to-ductal transition in *Kras^G12D^* mice. Apoptosis and immune cell infiltration occurred during acute pancreatitis, which might have influenced the serum miRNA profile.

### Serum miRNA-210, miRNA-21 and miRNA-29c levels are increased in *Kras^G12D^* mice without acute pancreatitis

**Figure 3:**
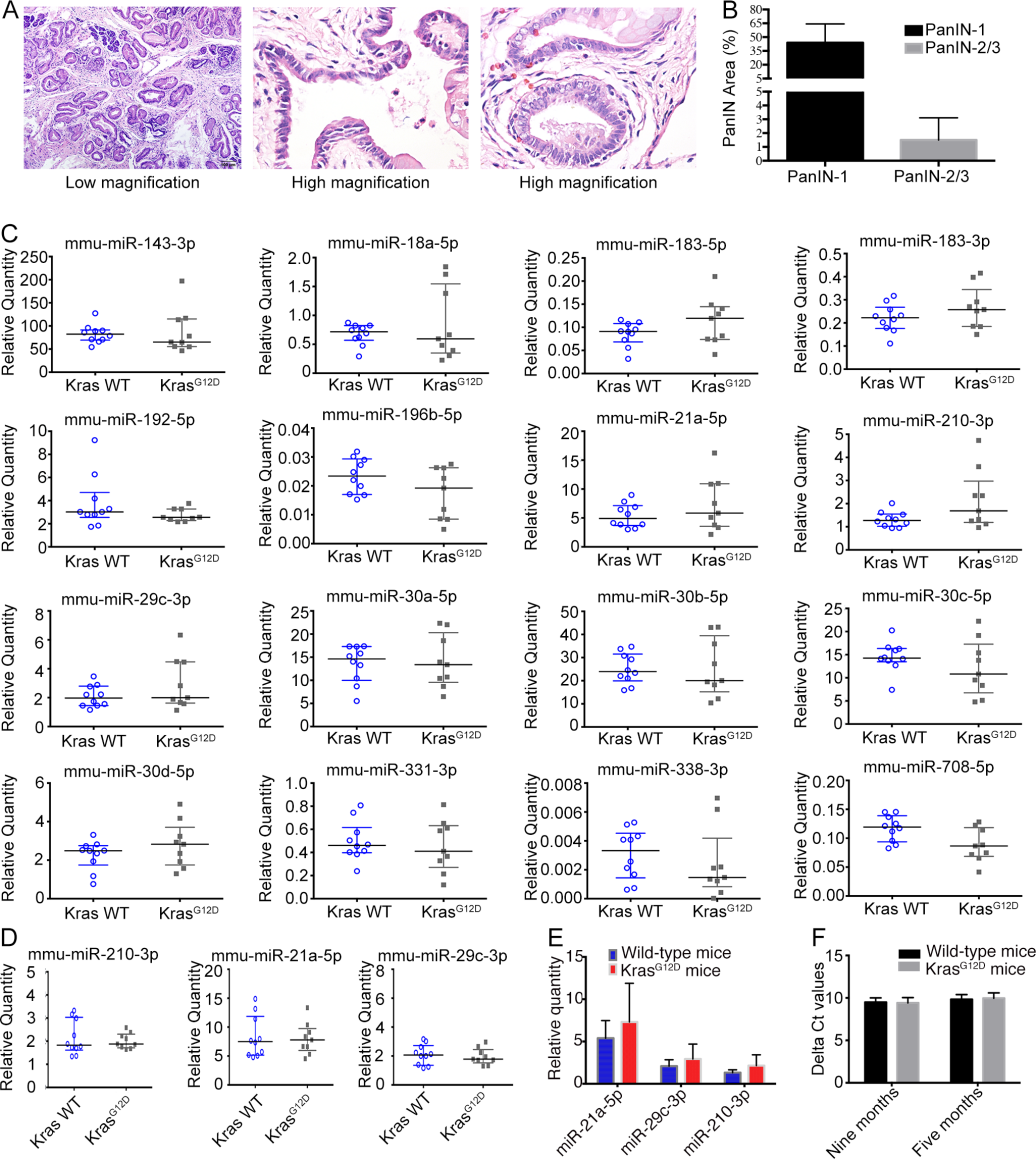
Serum miRNA in *Kras^G12D^* mice without acute pancreatitis (**A**) H & E staining of pancreas from *Kras^G12D^* mice without acute pancreatitis, middle/right panels showed high grade of PanIN (PanIN-2/3). (**B**) Quantification of PanIN-1 and PanIN-2/3. (**C**) MiRNA expression by realtime-PCR for 9-month-old mice. (**D**) MiRNA expression by realtime-PCR for 5-month-old mice. (**E**) Relative quantities for three increased miRNAs at 9-month of age. (**F**) Delta Ct value the two spike-in control miRNAs.

Nine *Kras^G12D^* mice without pancreatitis and ten age-matched wild-type mice were subjected to serial blood collection at 5 and 9 months of age. The mice were sacrificed at approximately 11 months of age; widespread PanIN-1 (Figure 3A, left panel) and occasional PanIN-2/3 (Figure 3A, middle and right panels) lesions were present in the *Kras^G12D^* mice. The quantification of PanIN-1 and PanIN-2/3 lesions is shown in Figure 3B.

The relative expression of the serum miRNAs was analysed with synthetic cel-miR-39 and cel-miR-54 spike-in controls; the average Ct values of these controls were used for serum miRNA quantification. For serum miRNA validation, a set of 16 miRNAs was quantified using serum samples from 9-month-old mice; ten miRNAs were increased in serum from *Kras^G12D^* mice with acute pancreatitis, and six miRNAs were previously reported to be highly expressed in PanIN or PanIN-3 tissue (Figure 3C) [16]. Three miRNAs (mmu-miR-21, mmu-miR-29c-3p and mmu-miR-210-3p) showed the highest fold changes in the *Kras^G12D^* group. To analyse the miRNA expression trends, we quantified the serum levels of mmu-miR-21, mmu-miR-29c-3p and mmu-miR-210-3p in 5-month-old mice but found no significant increases (Figure 3D). Therefore, certain serum miRNAs tended to increase in *Kras^G12D^* mice without acute pancreatitis in an age-dependent manner (Figure 3E); however, the spectrum of increased miRNAs differed in *Kras^G12D^* mice with and without acute pancreatitis. To validate that the system could reliably quantify miRNAs, the input quantity of cel-miR-54 was 1,000-fold higher than that of cel-miR-39, and we quantified the relative expression of cel-miR-54 normalized to cel-miR-39 as an internal control. The delta Ct value of cel-miR-54 was not significantly different between the *Kras^G12D^* and wild-type groups at either 5 or 9 months (Figure 3F).

### Human homologues of the increased serum miRNAs would be increased or decreased in human pancreatitis and pancreatic cancer tissue

Because the increased miRNAs likely originated from the pancreas, we were interested in how these miRNAs change during human pancreatitis and pancreatic cancer. Twenty-two of the 30 increased miRNAs have a homologous mature human miRNA (Table 1). To explore the expression pattern of these miRNAs in PanIN, pancreatic cancer, and pancreatitis tissue samples, we compared their relative tissue expression in micro-dissected PanIN cells and normal ductal cells [16]. Four human homologous mature miRNAs were significantly increased in PanIN cells, and one human homologous mature miRNA was significantly increased in PanIN-3 cells. In addition, we retrieved the relative miRNA levels in 136 pancreatic cancer, 27 pancreatitis and 22 normal pancreas tissue samples from a publicly available dataset (GSE24279). The miRNA expression changes trended in the same direction for both pancreatitis and pancreatic cancer. Eight miRNAs showed significantly higher levels in pancreatic cancer tissue compared with normal tissue; four of these eight also showed significantly higher levels in pancreatitis tissue compared with normal pancreas tissue. In contrast, ten miRNAs showed significantly lower levels in pancreatic cancer tissue compared with normal tissue, and five of these also showed significantly lower expression in pancreatitis tissue compared with normal pancreas tissue. The other four miRNAs showed no significant changes between pancreatic cancer and normal control tissue (Table 1). Nearly half of the miRNAs tended to change in the same direction for pancreatic cancer and pancreatitis, making it difficult to identify miRNA biomarkers specific for PanIN lesions. To test the possibility of interference from miRNA from residual blood cells, the expression of these 22 miRNAs was compared with that in a previous publication [17]; however, data were available for only 10 of these 22 miRNAs (Table 1).

**Table 1:** Serum up-regulated miRNAs for PanIN-pancreatitis status, expressions in PanIN cell, normal pancreas, pancreatitis and pancreatic cancer tissues, and expression in blood cells

MiRNA names	Expression fold changes in PanIN tissue	Relative expression			*P* values			Highly expressed from residual blood cells
		PC	Pancreatitis	Normal	PC versus normal	Pancreatitis versus Normal	PC versus Pancreatitis	
mmu-miR-200a-3p	6.8 (PanIN)	231.98	196.96	700.64	2.42E−05	1.07E−05	4.39E−01	No
**mmu-miR-192-5p**		310.20	450.18	1573.98	5.69E−09	6.01E−08	1.54E−01	N/A
**mmu-miR-30c-5p**		1089.63	2475.51	3048.49	3.66E−06	2.66E−01	1.96E−03	Yes
**mmu-miR-30b-5p**		762.23	1677.68	2133.47	1.57E−07	1.57E−01	1.74E−03	Yes
**mmu-miR-331-3p**	3.2 (PanIN)	309.38	217.04	136.55	1.12E−11	1.57E−02	6.77E−03	N/A
mmu-miR-375-3p		3983.12	6933.41	9632.71	6.69E−07	2.27E−02	2.17E−03	No
mmu-miR-92a-3p		1094.64	554.96	354.13	4.51E−11	6.39E−02	3.30E−06	Yes
mmu-miR-139-5p		92.26	60.94	65.53	2.99E−01	8.55E−01	2.30E−01	N/A
**mmu-miR-30a-5p**		1863.98	4525.40	5105.85	1.95E−04	5.92E−01	2.96E−03	N/A
mmu-miR-132-3p		431.84	236.44	92.30	2.92E−10	5.13E−03	1.68E−03	N/A
**mmu-miR-143-3p**		1233.11	519.54	765.17	1.01E−02	1.94E−01	4.03E−05	N/A
mmu-miR-15b-5p	6.3 (PanIN)	1974.22	1351.96	748.90	9.20E−12	7.46E−03	4.67E−03	Yes
**mmu-miR-30d-5p**		4860.68	7818.43	7812.94	5.86E−05	9.95E−01	1.03E−03	Yes
mmu-miR-409-3p		108.04	48.45	25.35	1.51E−06	1.13E−01	1.46E−03	N/A
**mmu-miR-210-3p**		259.70	8.45	−8.40	1.05E−10	8.52E−02	1.31E−09	No
mmu-miR-328-3p		−48.93	−53.00	−57.92	1.34E−01	4.88E−01	3.96E−01	Yes
mmu-miR-532-3p		90.03	87.80	21.62	1.32E−06	1.69E−03	9.03E−01	N/A
**mmu-miR-29c-3p**	6.7 (PanIN)	311.82	402.50	952.36	7.86E−07	1.91E−05	1.81E−01	N/A
mmu-miR-340-5p		−37.53	−50.27	−31.49	4.50E−01	2.90E−02	1.02E−02	N/A
mmu-miR-365-3p		64.26	91.09	227.95	9.26E−04	5.00E−03	8.51E−02	N/A
mmu-miR-206-3p		−48.82	−36.75	−36.13	1.73E−02	9.25E−01	2.42E−02	No
**mmu-miR-196b-5p**	64.9 (PanIN-3)	−49.97	−38.75	−46.81	5.67E−01	3.26E−01	1.03E−01	N/A

**Note:** PC: pancreatic cancer.

## DISCUSSION

It would be reasonable to screen for pre-malignant pancreatic lesions in patients presenting with pancreatitis symptoms because this population is at high risk for pancreatic cancer [6]. However, current guidelines do not recommend invasive procedures for most pancreatitis patients, making it difficult to know *a priori* which subpopulation of acute pancreatitis patients already harbours PanIN lesions. Thus, we used a *Kras^G12D^* mouse model to assess the practicality of distinguishing the presence or absence of PanIN in mice with acute pancreatitis. The data indicated that serum miRNAs cannot serve as sensitive biomarkers for detecting PanIN lesions in the setting of acute pancreatitis. Immune cell infiltration and the transition from acinar cells to ductal-like cells were the two sources of miRNA changes, which hindered the identification of miRNA alterations stemming solely from PanIN lesions.

We also tested whether certain serum miRNAs could distinguish mice with PanIN in the absence of acute pancreatitis from those with a normal healthy pancreas, since miRNA alternations did occur in *Kras^G12D^* mice pancreas tissue [18]. Three miRNAs showed a trend of higher levels in the PanIN group. MiR-210 is involved in the pathological progression of pancreatic cancer cells [19] and hypoxia acclimation [20], resulting from the extensive fibrosis observed during pancreatic cancer progression. Furthermore, patient-based serum miRNA studies showed increased levels of miR-21 and miR-210 in pancreatic cancer patients [21]. The fold changes in these two miRNAs in mice with PanIN in our study were lower than those in pancreatic cancer patients [21], which corresponds with the lower tumour burden associated with PanIN than with pancreatic cancer. MiR-29c, a tumour suppressor miRNA in pancreatic cancer [22–24], is increased in PanIN lesions [16] but significantly decreased in pancreatic cancer tissue [22, 24].

Some technical issues are important to note for future miRNA studies. First, serum lacks stably expressed endogenous control miRNAs. Synthetic *Caenorhabditis elegans* miRNAs have been used as quantification controls and have performed well in other studies [25]. The present study used cel-miR-39 and cel-miR-54 as external controls with a 1,000-fold difference in input quantity to verify the experimental accuracy. Second, serum/plasma samples are likely to contain residual miRNAs from blood cells [17, 26], and we strictly followed a protocol to minimize haemolysis during serum preparation. Four of the 22 increased miRNAs in *Kras^G12D^* mice with acute pancreatitis, including miR-210-3p, are not likely to have been affected by miRNAs from residual blood cells. Third, the *Kras^G12D^* mice without caerulein-treatment only contained less than 3% of PanIN-2/3 lesions at 11-month of age. Thus, certain PanIN-2/3 specific miRNAs would not be detectable when *Kras^G12D^* mice were less than 11-month of age in our experiment settings. Fourth, we did not address whether serum miRNA changes progressively in wild-type mice with various time lengths of caerulein treatment in this study, therefore, the different time lengths of caerulein treatment of *Kras^G12D^* and wild-type mice might influence the results of serum miRNA alteration.

In conclusion, the present data reveal that pancreatitis might interfere with serum miRNA profiling and thus make it difficult to use this tool for the early diagnosis of PanIN. Future studies should focus on *Kras^G12D^* mice without acute pancreatitis. New quantification techniques, such as miRNA sequencing, would be helpful for discovering new biomarkers for PanIN lesions.

## MATERIALS AND METHODS

### Animal model inbreeding

The animals were inbred according to animal ethics approval (No. NRCMM07), and all the experiments were conducted according to animal welfare guidelines [27]. B6.129-Krastm4Tyj/Nci (abbreviated as LSL-*Kras^G12D^*; strain number: 01XJ6) and B6.FVB-Tg(Ipf1-cre)1Tuv/Nci (abbreviated as Pdx1-cre; strain number: 01XL5) mice were imported from the National Cancer Institute (NCI) Mouse Repository. These strains were donated to the repository by Dr. Tyler Jacks, Massachusetts Institute of Technology, and Dr. Andrew M. Lowy, University of Cincinnati, respectively. The LSL-*Kras^G12D^*- and Pdx1-Cre-positive mice were named ‘*Kras^G12D^* mutant mice’ because they harbour oncogenic *Kras^G12D^* in the tail and pancreas and spontaneously develop PanIN lesions [28]. Caerulein (Sigma-Aldrich, St. Louis, MO, USA, Cat# C9026) was intra-abdominally injected to induce pancreatitis in all mice and to accelerate PanIN progression in experimental mice [29]. The mice were housed in a 12/12-hour light/dark cycle. All the animals were backcrossed to C57BL/6J mice (B6J, 000664) and then mated with other mice in the same cage. Genotyping was conducted using primers for both LSL-*Kras^G12D^* and Pdx1-Cre (Supplementary Table 3). The mice of other genotypes were named ‘control mice’. Caerulein was diluted in sterile distilled water to a final working concentration of 50 μg/ml and stored at −20°C until use. Caerulein working solution or placebo control was injected at a dose of 0.1 ml per day on Monday through Friday each week [29]. Mouse serum samples were collected by retro-orbital bleeding into Vacuette Serum Tubes (Cruinn Diagnostics, Dublin, Ireland, Cat# 454067), and the blood was incubated at room temperature for 30 minutes to allow for complete clotting. The tubes were then centrifuged at 2,000 × g for 15 minutes at 4°C to yield serum, which was frozen at −80°C until use.

### Histological grading and immunostaining of mouse pancreas tissue

Mouse pancreas tissues were fixed overnight in 4% paraformaldehyde (Sigma-Aldrich, Cat# 158127). Haematoxylin and eosin (H & E) staining and immunostaining were performed to analyse representative cross-sections of the pancreatic head, body and tail. Immunostaining was conducted according to standard protocols [30]. In brief, endogenous peroxide activity was blocked with 3% peroxidase, and the slides were then incubated with 5% BSA to prevent nonspecific binding. The slides were incubated with primary antibodies at an optimal dilution (clusterin: Santa Cruz Biotechnology, Cat# sc-8354, 1:100; cytokeratin 19: Abcam, Cat# ab15463, 1:100; chymotrypsin: Abcam, Cat# ab35694, 1:100; and amylase: Santa Cruz Biotechnology, Cat# sc-46657, 1:100) and appropriate secondary antibodies. The horseradish peroxidase polymer detection system (PV-9001; GBI Labs, Mukilteo, WA, USA) was employed to detect antibody staining. Five to ten randomly selected 100X H&E images were quantified by a pathologist using standard pathological criteria [31, 32].

### Pancreas mRNA quantification

Pancreas tissues were cut into small pieces and submerged overnight in RNAlater (Life Technologies, Cat# AM7022). The tissue samples were then frozen at −80°C until use. Briefly, total RNA was extracted from tissue with miRNeasy Mini Kit (QIAGEN,217004) and RNase-Free Dnase Set (QIAGEN,79254). RNA quality was analysed by electrophoresis of 28S and 18S ribosomal RNA. Biotin-labelled cDNA was obtained from 250 ng of total RNA with the Gene AmpPCR System 9700 (Life Technologies). Following the manufacturer's instructions, 10 μg of cDNA was fragmented and hybridized to the GeneChip^®^ Mouse Genome 430 2.0 Array. The DNA chips were washed, stained using the GeneChip^®^ Fluidics Station 450 and scanned using the GeneChip^®^ Scanner 3000 7G (Affymetrix, Part # 00-0213).

### Quantification of serum miRNAs

### Spike-in control

Two synthetic *C. Elegans* miRNAs (*Cel-miRs*), Cel-miR-39-3p (Life Technologies, Cat# MC10956) and Cel-miR-54-3p (Life Technologies, Cat# MC10279), were used as spike-in controls and were diluted in UltraPure DEPC-treated water (Life Technologies, Cat# MC 750023) to the working concentration. Five microlitres of each *Cel-miR* was added into 100 μL of a serum sample (Supplementary Table 4). These two *Cel-miRs* are not homologous to known human or mouse miRNAs, and they were previously validated as effective external controls for serum miRNA quantification [25].

### RNA extraction

RNA was extracted according to the manufacturer's instruction and a previous report [25]. Specifically, QIAzol Lysis Reagent was added to the reaction system to inactivate endogenous serum RNases before spiking in two synthetic miRNA controls at the respective working concentrations. The rest of the steps were the same as those in the standard protocol.

### Reverse transcription

MiRNA primers consisted of either the universal Megaplex™ RT Rodent Pool A/B v3.0 (10X) (Life Technologies, Cat# 4399970/4444292) or a mixture of target miRNA primers (Life Technologies, Cat# 4427975). Briefly, miRNA primers, TaqMan MiRNA Reverse Transcription Reagent (Life Technologies, Cat# 4366596) and nuclease-free water were mixed in a final volume of 4.5 μl. Then, 3 μl of total RNA was added to the reaction system, and 40 cycles of reverse transcription (16°C for 2 minutes, 42°C for 1 minute and 50°C for 1 second) were performed, followed by incubation at 85°C for 5 minutes and subsequent cooling to 4°C.

### Pre-amplification

MiRNA pre-amplification primers consisted of either Megaplex™ PreAmp Primers, Rodent Pool A/B v3.0 (Life Technologies, Cat# 4399203/4444308) or Megaplex PreAmp Primers (Life Technologies, Cat# 4444747). The pre-amplification reactions included cDNA from the previous step, TaqMan PreAmp Master Mix (Life Technologies, Cat# 4391128), miRNA pre-amplification primers and nuclease-free water and were performed at 95°C for 10 minutes, 55°C for 2 minutes, and 72°C for 2 minutes, followed by 12 cycles of 95°C for 15 seconds and 60°C for 4 minutes. The pre-amplification products were diluted in DEPC-treated water.

### MiRNA expression profiling

The PCR samples contained the following: diluted pre-amplified cDNA, TaqMan Universal Master Mix II without uracil N-glycosylase (UNG) (Life Technologies, Cat# 4440040) and nuclease-free water. The reactions were performed in a 7900HT machine with a System Automation Accessory Upgrade (Life Technologies, Cat# 4329007). The 2 cards were designed with 768 assays for miRNAs from Sanger miRBase v14.

### Real-time PCR for individual miRNA targets

The PCR mixtures contained the following: cDNA product, TaqMan Universal Master Mix II without UNG, 20× TaqMan MiRNA Assays, and nuclease-free water. PCR was performed at 94.5°C for 10 minutes, followed by 40 reaction cycles of 97°C for 30 seconds and 59.7°C for 1 minute, in a 7900HT Fast Real-Time PCR Machine (Life Technologies, Cat# 4351405).

### Statistical analysis

The pancreas mRNA expression microarray data were normalized using the Robust Multi-array Average (RMA) method [33], and GSEA was conducted according to the standard protocol to identify enriched pathways [34]. The pancreas mRNA expression microarray data were processed with the ‘affy’ package [35]. The normalized values from mice with or without caerulein treatment were compared using the ‘limma’ package [36]. The heatmap was generated using the ‘gplots’ package [37], with clustering of the selected genes.

Serum miRNA profiling data were normalized using both VSN [38] and invariant methods [39]. For the VSN method, the amount of miRNA(x) with a Ct value of x was calculated as 2^[50–Ct(x)]^, in which an miRNA with a Ct value of 50 was arbitrarily defined as one unit to facilitate calculation. For the invariant method, we chose “mmu-miR-146a-5p”, “mmu-miR-16-5p”, “mmu-miR-30e-5p” and “mmu-miR-744-5p” as invariant serum miRNAs, as indicated in a previous study [40]. The heatmap was generated using the ‘gplots’ package [37] with hierarchical clustering of all miRNAs with available values or of the significantly changed miRNAs.

Tissue miRNA expression profiles were normalized using U6 snRNA as an endogenous control. The normalized Ct value of miRNA(x) was 2^[Ct(U6 snRNA)–Ct(x)]^ [41]. MiRNA expression was ranked based on the average normalized Ct value of duplicate pancreas tissue samples.

For miRNA real-time PCR, the relative expression of miRNA(x) was calculated as 2^[average[Ct(cel-miR-39-3p) and Ct(cel-miR-54-3p)]–Ct(x)]^. Individual miRNA real-time PCR data were normalized by standard methods.

Student's two-tailed *t* test was conducted with the assumption of unequal standard deviations. Statistical significance was set at *p* < 0.05. The kappa index was according to the standard calculation method [42]. All the analyses were conducted using R 3.1.0 and GraphPad Prism version 6 for Mac OS X (GraphPad Software, La Jolla, CA, USA).

## SUPPLEMENTARY MATERIALS TABLES


