# Supplementary material for: Challenges in detecting pre-malignant pancreatic lesions during acute pancreatitis using a serum microRNA assay: a study based on KrasG12D transgenic mice
Source: Oncotarget. 2016 Mar 17;7(16):22700–10. doi: 10.18632/oncotarget.8148 (PMC5008393; doi:10.18632/oncotarget.8148)
Supplement: Supplementary file 1 [file oncotarget-07-22700-s001.pdf]

Challenges in detecting pre-malignant pancreatic lesions during acute pancreatitis using a serum microRNA assay: a study based on *Kras*^{G12D} transgenic mice

Supplementary Materials

Supplementary Table S1: Increased/decreased miRNA between *Kras*^{G12D} mice with acute pancreatitis and wild-type mice without pancreatitis

MiRNA names	VSN method				Invariant method				Up/Down regulation
	mean_control_VSN	mean_case_VSN	delta_mean_VSN	*p*_value_VSN	mean_control_invariant	mean_case_invariant	delta_mean_invari-ant	*p*_value_invariant	
mmu-miR-138-1-3p	20.038	24.196	4.158	0.045	70.266	74.339	4.074	0.039	Up
mmu-miR-547-3p	19.075	22.505	3.431	0.019	69.302	72.649	3.346	0.029	Up
mmu-miR-182-5p	25.808	29.217	3.408	0.015	76.036	79.360	3.324	0.036	Up
mmu-miR-375-3p	26.689	30.019	3.33	0.004	76.917	80.162	3.246	0.010	Up
rno-miR-381	18.095	21.374	3.279	0.01	68.322	71.517	3.195	0.018	Up
mmu-miR-196b-5p	24.356	27.598	3.241	0.008	74.584	77.741	3.157	0.011	Up
mmu-miR-132-3p	27.031	29.505	2.473	0.025	77.259	79.648	2.389	0.049	Up
mmu-miR-29c-3p	28.777	31.219	2.442	0.002	79.004	81.362	2.358	0.006	Up
mmu-miR-434-3p	25.178	27.568	2.39	0.011	75.406	77.711	2.306	0.038	Up
mmu-miR-409-3p	24.104	26.099	1.994	0.008	74.332	76.242	1.910	0.027	Up
mmu-miR-215-5p	28.826	30.797	1.971	0.001	79.054	80.941	1.887	0.009	Up
mmu-miR-34b-3p	24.649	26.52	1.871	0.002	74.877	76.664	1.787	0.016	Up
mmu-miR-200a-3p	30.6	32.384	1.784	0.024	80.828	82.528	1.700	0.016	Up

mmu-miR-365-3p	25.975	27.727	1.752	0.000	76.203	77.870	1.668	0.005	Up
mmu-miR-15b-5p	32.619	34.342	1.723	0.001	82.846	84.485	1.639	0.001	Up
mmu-miR-328-3p	32.089	33.751	1.662	0.005	82.316	83.894	1.578	0.039	Up
mmu-miR-192-5p	31.018	32.634	1.617	0.009	81.246	82.778	1.532	0.038	Up
mmu-miR-210-3p	30.34	31.934	1.594	0.005	80.568	82.078	1.510	0.011	Up
rno-miR-224	22.256	23.813	1.556	0.011	72.484	73.956	1.472	0.046	Up
mmu-miR-30d-5p	30.611	32.118	1.506	0.001	80.839	82.261	1.422	0.006	Up
mmu-miR-143-3p	29.073	30.558	1.485	0.019	79.300	80.701	1.401	0.037	Up
mmu-miR-30c-5p	35.599	36.969	1.37	0.004	85.827	87.112	1.285	0.043	Up
mmu-miR-206-3p	30.631	31.943	1.312	0.016	80.858	82.086	1.228	0.005	Up
mmu-miR-139-5p	30.648	31.814	1.166	0.005	80.875	81.957	1.082	0.047	Up
mmu-miR-30b-5p	35.513	36.647	1.134	0.026	85.741	86.791	1.050	0.012	Up
mmu-miR-92a-3p	35.532	36.497	0.965	0.009	85.760	86.641	0.881	0.002	Up
mmu-miR-532-3p	28.184	29.078	0.894	0.007	78.412	79.221	0.809	0.033	Up
mmu-miR-331-3p	30.625	31.468	0.843	0.035	80.853	81.612	0.759	0.001	Up
mmu-miR-340-5p	28.705	29.522	0.817	0.03	78.932	79.665	0.733	0.025	Up
mmu-miR-30a-5p	33.726	34.419	0.693	0.033	83.954	84.562	0.609	0.012	Up
mmu-miR-532-5p	30.644	30.16	−0.484	0.027	80.872	80.304	–0.568	0.044	Down
rno-miR-7a#	30.081	29.285	–0.796	0.004	80.309	79.428	–0.881	0.022	Down
mmu-miR-193-3p	21.15	20.207	–0.943	0.02	71.378	70.350	–1.027	0.010	Down
mmu-miR-186-3p	26.027	25.033	–0.993	0.039	76.254	75.177	−1.078	0.006	Down

mmu-miR-195-5p	34.56	33.488	−1.072	0.021	84.788	83.631	−1.156	0.009	Down
mmu-miR-2134(15)	37.493	36.283	−1.21	0.017	87.721	86.427	−1.295	0.036	Down
mmu-miR-542-5p	22.177	20.96	−1.217	0.023	72.405	71.104	−1.302	0.043	Down
mmu-let-7a-1-3p	24.377	23.153	−1.224	0.011	74.605	73.296	−1.309	0.034	Down
rno-miR-345-3p	24.607	23.351	−1.256	0.021	74.835	73.494	−1.341	0.013	Down
mmu-miR-872-3p	26.672	25.332	−1.341	0.001	76.900	75.475	−1.425	0.025	Down
mmu-miR-15b-3p	30.606	29.197	−1.409	0.016	80.833	79.340	−1.493	0.001	Down
hsa-miR-136#	23.855	22.367	−1.488	0.029	74.082	72.510	−1.572	0.047	Down
mmu-miR-297a-3p	24.181	22.644	−1.537	0.003	74.409	72.788	−1.621	0.039	Down
mmu-miR-425-3p	26.158	24.602	−1.556	0.001	76.385	74.745	−1.640	0.013	Down
mmu-miR-22-5p	25.618	24.052	−1.566	0.000	75.846	74.196	−1.650	0.003	Down
mmu-miR-1306-3p	24.5	22.915	−1.585	0.002	74.728	73.059	−1.669	0.009	Down
mmu-miR-322-3p	26.858	25.18	−1.679	0.01	77.086	75.323	−1.763	0.024	Down
mmu-miR-196a-5p	25.645	23.966	−1.68	0.008	75.873	74.109	−1.764	0.002	Down
mmu-miR-15a-3p	27.866	26.176	−1.69	0.006	78.094	76.319	−1.775	0.009	Down
rno-miR-352	25.952	24.245	−1.707	0.001	76.180	74.389	−1.791	0.012	Down
mmu-miR-2146(15)	32.786	31.052	−1.734	0.002	83.014	81.195	−1.819	0.000	Down
mmu-miR-21-3p	22.348	20.585	−1.763	0.016	72.575	70.728	−1.847	0.044	Down
mmu-miR-7b-5p	23.432	21.666	−1.766	0.004	73.660	71.810	−1.850	0.005	Down
mmu-miR-339-3p	26.5	24.674	−1.826	0.02	76.727	74.817	−1.910	0.038	Down
mmu-miR-450b-3p	23.587	21.754	−1.833	0.016	73.814	71.897	−1.918	0.041	Down

mmu-miR-1954	23.039	21.15	−1.889	0.000	73.267	71.294	−1.973	0.003	Down
mmu-miR-1949	25.923	23.876	−2.047	0.005	76.150	74.019	−2.131	0.038	Down
mmu-let-7g-3p	21.492	19.415	−2.076	0.000	71.719	69.558	−2.161	0.000	Down
mmu-let-7i-3p	21.596	19.437	−2.158	0.012	71.823	69.581	−2.243	0.005	Down
mmu-miR-338(9.2)	22.952	20.79	−2.162	0.002	73.180	70.934	−2.246	0.021	Down
mmu-miR-1944(16)	27.203	25.033	−2.17	0.022	77.430	75.176	−2.254	0.020	Down
mmu-miR-4 94(9.2)	17.149	14.94	−2.209	0.016	67.377	65.084	−2.293	0.013	Down
mmu-miR-449c-5p	25.258	22.855	−2.403	0.003	75.486	72.998	−2.488	0.000	Down
mmu-miR-1953	20.078	17.668	−2.41	0.013	70.306	67.812	−2.494	0.005	Down
mmu-miR-214-5p	25.941	23.486	−2.455	0.013	76.169	73.629	−2.540	0.025	Down
mmu-miR-99b-3p	21.831	19.328	−2.503	0.004	72.059	69.472	−2.587	0.017	Down
mmu-miR-1950	19.279	16.733	−2.546	0.014	69.507	66.876	−2.631	0.026	Down
mmu-miR-467b-3p	22.441	19.861	−2.58	0.026	72.669	70.004	−2.665	0.032	Down
mmu-miR-26b-3p	23.085	20.496	−2.589	0.005	73.313	70.639	−2.674	0.016	Down
mmu-miR-669m-3p	22.675	20.053	−2.622	0.000	72.903	70.197	−2.706	0.000	Down
mmu-miR-10b(9.2)	20.493	17.801	−2.693	0.004	70.721	67.944	−2.777	0.004	Down
mmu-miR-1932	19.903	17.203	−2.7	0.023	70.131	67.346	−2.785	0.037	Down
mmu-miR-1968-5p	22.737	19.93	−2.807	0.008	72.965	70.073	−2.892	0.032	Down
rno-miR-99a#	21.521	18.645	−2.876	0.003	71.748	68.788	−2.960	0.001	Down
mmu-miR-455-5p	25.69	22.136	−3.555	0.004	75.918	72.279	−3.639	0.002	Down
mmu-miR-1947-5p	21.742	17.712	−4.03	0.001	71.969	67.855	−4.114	0.000	Down

Supplementary Table S2: Increased/decreased miRNA between *Kras*^{G12D} mice with acute pancreatitis and wild-type mice with pancreatitis

MiRNA names	Invariant method				VSN method				Up/Down regulation
	mean_control_invariant	mean_case_invariant	delta_mean_invariant	*p*_value_invariant	mean_control_VSN	mean_case_VSN	delta_mean_VSN	*p*_value_VSN	
mmu-miR-293-3p	72.488	65.038	7.450	0.001	22.321	15.055	7.266	0.001	Down
**mmu-miR-196b-5p**	79.010	74.804	4.206	0.002	28.843	24.821	4.022	0.003	Down
mmu-miR-466j	74.602	72.651	1.951	0.014	24.435	22.667	1.768	0.018	Down
mmu-miR-214(9.2)	80.798	79.438	1.360	0.026	30.631	29.455	1.176	0.014	Down
mmu-miR-223(9.2)	89.686	88.393	1.293	0.022	39.520	38.410	1.109	0.019	Down
**mmu-miR-210-3p**	82.400	81.380	1.020	0.016	32.234	31.397	0.837	0.043	Down
rno-miR-532-5p	76.798	77.766	−0.968	0.031	26.632	27.783	−1.151	0.007	Up
mmu-miR-345-5p	76.796	77.826	−1.031	0.038	26.629	27.843	−1.214	0.007	Up
**mmu-miR-1944(16)**	76.905	77.967	−1.062	0.026	26.739	27.984	−1.245	0.048	Up
mmu-miR-539-5p	75.261	76.464	−1.203	0.029	25.094	26.481	−1.387	0.038	Up
**mmu-miR-542-5p**	74.783	76.884	−2.101	0.035	24.617	26.901	−2.284	0.023	Up

Supplementary Table S3: Primer sequences for genotyping the LSL-Kras and Pdx1-Cre loci

Gene	Forward Primer	Reverse Primer
LSL-KrasG12D	CCTTTACAAGCGCACGCAGACTGTAGA	AGCTAGCCACCATGGCTTGAGTAAGTCTGC
Pdx-Cre	CTGGACTACATCTTGAGTTGC	GGTGTACGGTCAGTAAATTTG

Supplementary Table S4: Spike-in controls for serum miRNA real-time PCR assay

Cel-miR	Sequence	Molecular Weight (g/mole)	nmole/OD260	μg/OD260	Working Concentration	Amount/100 ul serum	Concentration in serum
Cel-miR-39-3p (ssDNA)	5′-UCACCGGGU GUAAAUCAG CUUG-3′	6666.3	4.9	32.69	4 fmol/μL	20 fmol	200 fmol/ml
Cel-miR-54-3p (dsDNA)	5′-UACCCGUAAU CUUCAUAAUCC-GAG-3	14667.4	2.14	31.44	4 pmol/μL	20 pmol	200 pmol/ml
